# Effect of remote ischemic postconditioning on hepatic ischemia–reperfusion injury in patients undergoing laparoscopic hepatectomy: a randomized double-blinded controlled trial

**DOI:** 10.3389/fmed.2025.1628781

**Published:** 2025-09-26

**Authors:** Chao Yang, Lijun Yang, Mingwang Zeng, Yunyan Zhu, Chuanwu Zhang, Jing Chen, Yi Wang, Jinji Peng, Li-feng Wang, Maolin Zhong, Haiyu Xie, Weidong Liang

**Affiliations:** ^1^First Clinical Medical College of Gannan Medical University, Ganzhou, China; ^2^Department of Anesthesia Surgery Center, First Affiliated Hospital of Gannan Medical University, Ganzhou, China

**Keywords:** remote ischemic postconditioning, hepatectomy, ischemia–reperfusion injury, liver function, laparoscopic surgery

## Abstract

**Background:**

Hepatic ischemia–reperfusion injury (HIRI) remains a major determinant of outcomes after hepatectomy with hepatic portal occlusion. Animal studies suggest that remote ischemic postconditioning (RIPostC) could alleviate HIRI, but its clinical value is unclear. This trial aimed to evaluate the efficacy of RIPostC in patients undergoing laparoscopic hepatectomy.

**Methods:**

In this randomized controlled trial, 83 patients were assigned to receive either RIPostC (4 cycles of 5 min ischemia/5 min reperfusion) or no intervention. The primary endpoints were postoperative liver function biochemical markers in the serum [alanine aminotransferase (ALT), aspartate aminotransferase (AST), and total bilirubin (TBIL) levels] on postoperative days 1 and 2. The secondary outcomes included intraoperative variables and hospital stay.

**Results:**

Data from a total of 72 patients (36 per group) were analyzed. RIPostC did not significantly reduce ALT, AST or TBIL compared with control group (all *p* > 0.05). No differences were observed in Pringle time, operation time or length of postoperative hospital stay (*p* > 0.05).

**Conclusion:**

This study represents one of the first randomized controlled clinical evaluations of RIPostC during laparoscopic hepatectomy. Contrary to experimental findings, a single RIPostC protocol did not improve early postoperative liver function.

## Introduction

Liver cancer and intrahepatic bile duct stones are the most common liver diseases worldwide. According to global cancer statistics, the incidence of liver cancer continues to rise, with China alone accounting for about half of the global burden (1, 2). Hepatectomy remains the primary treatment for both benign and malignant liver diseases (2, 3). However, excessive intraoperative bleeding during liver parenchymal transection is still a major challenge (4). The Pringle maneuver is commonly used to reduce intraoperative bleeding, but inevitably leads to hepatic ischemia–reperfusion injury (HIRI), a critical postoperative complication that can progress to liver failure, significantly increasing morbidity and mortality after hepatectomy (5–7). Post-hepatectomy liver failure occurs in approximately 8–12% of patients, significantly increasing the risk of postoperative infections, cardiovascular events, and multiple organ failure, contributing to reduced long-term survival, and substantial healthcare care costs (8–12). Consequently, developing effective strategies to mitigate HIRI and improve postoperative outcomes remains an urgent priority in hepatobiliary surgery.

The pathogenesis of HIRI involves multiple complex mechanisms. During ischemia, hepatocytes suffer from impaired energy metabolism, and abnormal accumulation of intracellular metabolites. Subsequent reperfusion restores blood flow, but provokes oxidative stress, release of pro-inflammatory cytokines, and immune cell infiltration, leading to hepatocyte death through apoptosis, necrosis, and ferroptosis (7, 8, 13). These processes contribute to postoperative liver dysfunction and increase the risk of severe postoperative complications such as liver failure (10).

Various strategies have been explored to alleviate the physiological damage caused by HIRI, including pharmacological interventions, ischemic preconditioning, and machine perfusion techniques, such as hypothermic oxygenated perfusion (14, 15). However, many of these approaches remain in the preclinical stage. Remote ischemic preconditioning (RIPC), which involves repeated transient ischemia–reperfusion cycles in distant organs, has shown potential to enhance hepatic tolerance to ischemia (16, 17). Nevertheless, its clinical use is hindered by timing and standardization issues. To address these limitations, Kerendi et al. (18) introduced remote ischemic postconditioning (RIPostC) in 2005, demonstrating that brief, intermittent renal ischemia–reperfusion at the onset of re-perfusion significantly reduced myocardial infarct size in rats. Since then, RIPostC has been recognized as a simple, non-invasive cost-effective strategy that activates endogenous protective pathways. Experimental studies in the heart and brain have shown that RIPostC attenuates HIRI by reducing oxidative stress and inflammatory response, inhibiting apoptosis, improving hepatic sinus micro-circulation (19, 20). In the animal study of rat liver ischemia–reperfusion model, RIPostC can effectively reduce HIRI and improve postoperative liver function (21–23). Recent advances also highlight its pharmacological, immunological, and therapeutic potential.

Despite encouraging preclinical findings, clinical evidence for RIPostC in liver surgery remains scarce. To bridge this knowledge gap, we conducted a randomized controlled trial to evaluate the protective effects of RIPostC on HIRI in patients undergoing laparoscopic hepatectomy. By providing clinical evidence, this study aims to determine whether RIPostC can serve as a feasible perioperative strategy to mitigate liver HIRI, reduce postoperative complications, and improve patient recovery in hepatobiliary surgery.

## Materials and methods

### Ethical approval

This randomized, intervention-controlled clinical trial was conducted in the First Affiliated Hospital of Gannan Medical University of Jiangxi Province. The study protocol was approved by the hospital’s Ethics Committee (Approval No. LLSC-2022111801) and complied with the principles of the Helsinki Declaration and the CONSORT guidelines. The trial was registered in the Chinese Clinical Trial Registry (Registration No. ChiCTR2200066202). Written informed consent form was obtained from all participants or their legal representatives, and patient recruitment was completed by anesthesiologist.

### Participants

This was a single-center, randomized controlled trial. From December 2022 to November 2023, 72 patients scheduled for elective or time-limited laparoscopic hepatectomy with hepatic portal occlusion were enrolled according to the predefined inclusion and exclusion criteria. The inclusion criteria were as follows: (1) Age 18 to 75 years, scheduled for laparoscopic partial hepatectomy; (2) Intact cognitive function; no contraindications to surgery or anesthesia; (3) American Society of Anesthesiologists (ASA) grade I ~ III; (4) Provision of written informed consent by the Patients or their families. The exclusion criteria were as follows:(1) local limb infection or dysfunction; (2) severe cardiovascular or cerebrovascular accident history or lung (predicted by forced expiratory volume in 1 s (FEV1) < 40%), liver (Child-Pugh C grade) or renal dysfunction (Glomerular Filtration Rate < 30 mL/min/1.73 m^2^); (3) peripheral vascular disease; uncontrolled hypertension, diabetes, or sepsis;(4) absence of hepatic portal occlusion during surgery; (5) conversion to open surgery or a change in surgical procedure; (6) intraoperative blood loss ≥ 15% of total blood volume or ≥ 3 times infusions of vasoactive drugs during the operation, with a mean arterial pressure (MAP) ≤ 60 mmHg.

During liver parenchymal transection using an ultrasonic scalpel, all patients underwent standard hepatic surgical blood flow occlusion with the pringle maneuver: surgeons applied a latex tape to clamp the hepatic pedicle until the distal artery pulsation ceased. Each clamping time did not exceed 15 min, followed by 10 min of reperfusion.

### Sample size calculation

Sample size estimation was based on prior studies. In one trail, the Remote ischemic preconditioning (RIPC) group had significantly lower serum ALT levels (41%) than controls (412 ± 144 vs. 698 ± 137 IU/L; *p* = 0.026) (16). We conservatively assumed a 15% effect size, with a mean intergroup difference of 105, standard deviation 137, a two-sided *α* = 0.05, and the statistical power (1-*β*) = 0.9. According to the following sample size calculation formula, the sample size of each group was calculated to be 36 patients. Considering a 15% dropout rate, a total of 87 patients were ultimately recruited.

### Randomization and blindness

A computer-generated randomization sequence was created using SPSS 26.0 software by an independent statistician not involved in patient recruitment or data collection. Block randomization was used to ensure a balanced allocation of participants between the RIPostC group and the control group in a 1:1 ratio, with 36 patients in each group. Allocation concealment was maintained using sequentially numbered, opaque, sealed envelopes (SNOSE), prepared by an independent researcher not involved in participant enrollment. Envelopes were opened sequentially only after a participant’s eligibility had been confirmed and enrollment was completed. Only anesthesia nurses had access to these envelopes and administered the assigned interventions accordingly. To maintain blinding, the following precautions were taken: Surgeons, anesthesiologists, patients, and outcome assessors were blinded to group allocation; Research personnel responsible for postoperative follow-up and data analysis were independent of those involved in patient management; Electronic data access restrictions were implemented to prevent study personnel from viewing allocation details. After surgery, a researcher who did not participate in the preliminary study conducted postoperative follow-up and recorded the analysis data. The entire study process, from the initiation to completion was regularly supervised by a designed monitor to ensure protocol compliance.

### RIPostC intervention

The specific distal ischemic treatment was carried out by relevant research personnel through a limb ischemic preconditioning device (Shenzhen City, model RIP-809S). The relevant instrument was tied to the proximal third of the thigh, and the pressure was set at 200 mmHg (1 mmHg = 0.133 kPa), right lower limb ischemia was successful if the dorsalis pedis was absent. At the exact moment of release of hepatic portal occlusion and initiation of continuous reperfusion, 5 min of ischemia followed by 5 min of reperfusion was performed on the right lower limb of patients in the RIPostC group, which was repeated for 4 cycles for a total of 40 min. The same method was used for the control group—the instrument was placed at the same site but was not inflated.

### Anesthesia management

All patients received the same anesthetic protocol. Patients fasted for at least 8 h and abstained from drinking for 2 h before the operation. Upon arrival to the operating room, a peripheral venous line was established, and the following basic vital signs were monitored: electrocardiogram, blood oxygen saturation, and body temperature. Arterial blood pressure was continuously monitored invasively during the operation. Anesthesia was induced with midazolam (Jiangsu Enhua Pharmaceutical Co., Ltd., H19990027) 0.05 mg/kg, penehyclidine hydrochloride (Jingzhou Aohong Pharmaceutical Co., Ltd., H20020606) 0.01 mg/kg, etomidate (Jiangsu Enhua Pharmaceutical Co., Ltd., H20020511) 0.3 mg/kg, cisatracurium (Hangzhou Aoya Biotechnology Co., Ltd., H20213438) 0.2 mg/kg and sufentanil (Yichang Renfu Pharmaceutical Co., Ltd., H20054171) 0.5 μg/kg. Tracheal intubation was performed under a visual laryngoscope, and then the ventilator was connected, and mechanical ventilation was initiated. A double-lumen central venous catheter was inserted. Anesthesia was maintained with continuous intravenous infusions of 4~12 mg/kg·h propofol (Sichuan Guorui Pharmaceutical Co., Ltd., H20030115) and 0.1~0.3 μg/kg·min remifentanil (Yichang Renfu Pharmaceutical Co., Ltd., H20030197), and the infusion rate was adjusted to maintain the appropriate depth of anesthesia and intensity of surgical stimulation. Sufentanil and cis-atracurium were added intermittently according to the needs of the operation. At the end of the operation, the infusion of propofol and remifentanil was stopped immediately, and the analgesia pump was connected 10 min in advance. All procedures were performed by the same team of surgeons and anesthesiologists.

### Outcomes

The main observation indexes were serum levels of alanine aminotransferase (ALT), aspartate aminotransferase (AST) and total bilirubin (TBIL) on postoperative days 1 d and 2. The secondary observation indexes included anesthesia time, operation time and postoperative hospital stay. Three milliliters of venous blood was extracted from all patients at the above three time points, and the samples were centrifuged at 3,000 r/min for 15 min (Germany, model Heraeus Multifuge X1R). Then, the supernatant was stored in an ultralow temperature refrigerator at −80 °C (United States, model Forma 702). After all the samples were thawed, the biochemical indices of liver function in the supernatant were detected and recorded by an automatic biochemical analyzer to evaluate liver function (Germany, model Cobas 8000 Core).

### Protocol adherence and monitoring

To ensure protocol adherence and minimize deviations, a dedicated study monitor conducted weekly audits throughout the study. The audits included: Verification of proper sequence generation and envelope allocation; Random chart reviews to check compliance with treatment protocols; Direct observation of intervention administration in selected cases; Review of missing data and protocol deviations, with corrective actions taken as necessary.

### Adverse event monitoring and assessment of harms

Adverse events (AEs) and serious adverse events (SAEs) were monitored from enrollment to hospital discharge. The monitoring and reporting process followed the Common Terminology Criteria for Adverse Events (CTCAE) v5.0. AEs were categorized into mild, moderate, or severe, based on clinical impact. All AEs were recorded in case report forms (CRFs), and severe events were reported to the DSMC within 24 h. If an AE was suspected to be related to the intervention, the treating physician, DSMC, and Ethics Committee would determine whether the patient should be withdrawn from the study. An interim analysis was planned in case of unexpected high rates of AEs to determine if early study termination was warranted. All AEs were analyzed at the end of the study, including comparisons between groups to assess intervention safety. No specific harms were defined or assessed, as the intervention was considered low risk.

### Statistical analysis

All the data were analyzed with SPSS 26.0 software. Quantitative data are expressed as the mean ± standard deviation (^−^*x* ± s). Quantitative data were tested by Shapiro-Wilker test to confirm the normal distribution of data. A t test was used for comparisons between groups, and repeated measures analysis of variance was used for multiple time points. The remaining quantitative data are expressed as the median (P_50_) and interquartile range (P_25_, P_75_), and the nonparametric rank sum test was used. Qualitative data are expressed as the composition ratio or percentage (%). The chi-square test or Fisher’s exact test was used for comparisons between groups. A *p* value *<* 0.05 was considered to indicate statistical significance.

## Results

### Population characteristics

**Figure 1:**
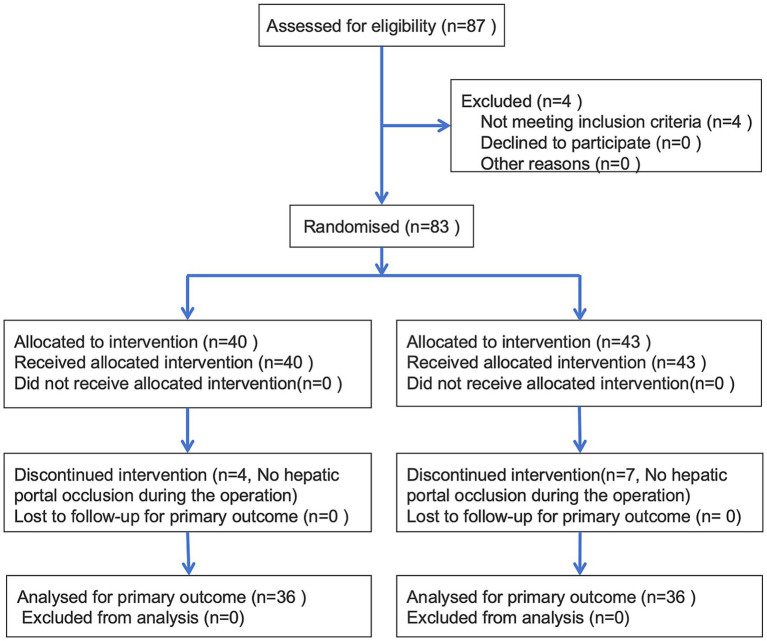
Study flow diagram of participants in the randomized trial.

A total of 87 patients with surgical indications for hepatectomy were assessed for eligibility. Of these, 15 patients were excluded due to the absence of hepatic portal occlusion or other reasons, leaving 72 patients for the final analysis. The study flowchart is shown in Figure 1.

Baseline demographic and clinical characteristics, including sex, age, weight, height, ASA score, cirrhosis, hypertension, and diabetes, were comparable between the two groups, with no statistically significant differences (all *p* > 0.05; Table 1).

**Table 1:** Characteristics of the patients.

Characteristics	Control group (*n* = 36)	RIPostC group (*n* = 36)	*p*-value
Sex (male)	20 (55.6%)	25 (69.4%)	0.22
Age (y)	52.28 ± 13.87	55.58 ± 12.63	0.93
Weight (kg)	59.78 ± 11.63	60.81 ± 9.15	0.27
Height (cm)	162.33 ± 8.73	162.11 ± 5.75	0.90
ASA score, I /II/III	9/24/3	13/22/1	0.41
Cirrhosis	20 (55.6%)	22 (61.1%)	0.63
Hypertension	7 (19.4%)	6 (16.7%)	0.76
Diabetes	3 (8.3%)	4 (11.1%)	1.00

Data are presented as mean ± SD, median (range), or *n* (%). ASA, American Society of Anesthesiologists; RIPostC, remote ischemic postconditioning; SD, standard deviation.

### Perioperative characteristics

Key perioperative variables such as Pringle time, liver resection time, surgical time, and anesthesia time were analyzed, as these factors may influence the extent of hepatic ischemia–reperfusion injury. However, no significant differences were observed between the two groups (all *p* > 0.05, Table 2).

**Table 2:** Perioperative data.

Characteristics	Control group (*n* = 36)	RIPostC group (*n* = 36)	*p*-value
Pringle (time)	2.00 (1.25,3.00)	3.00 (2.00,4.00)	0.33
Pringle time (min)	33.50 (19.00,47.75)	38.00 (31.00,55.00)	0.33
Liver resection time (min)	80.00 (70.00,110.00)	100.00 (70.00,120.00)	0.36
Surgical time (min)	177.50 (136.25,233.75)	192.50 (156.25,243.75)	0.69
Anesthesia time (min)	227.50 (196.25,285.00)	242.50 (201.25,297.50)	0.69
PACU time (min)	84.31 ± 24.06	80.69 ± 32.19	0.59
Operative procedure			0.67
Hemi-hepatectomy	7 (19.4%)	10 (27.8%)	
Segment resection	18 (50.0%)	15 (41.7%)	
Irregular resection	11 (30.6%)	11 (30.6%)	
Indications for resection			1.00
Benign	17 (47.2%)	17 (47.2%)	
Malignant	19 (52.8%)	19 (52.8%)	
Blood loss (mL)	325.56 ± 185.39	317.22 ± 177.90	0.85
Transfusion fluid (mL)	2183.33 ± 361.35	2191.67 ± 417.73	0.93
Urine output (mL)	400.00 (300.00,500.00)	375.00 (262.50,500.00)	0.83
p-RBC transfusion	1 (1.4%)	0 (0.0%)	1.00
Vasopressor	17 (47.2%)	13 (36.1%)	0.34
Postoperative hospital stay (day)	10.25 ± 3.83	10.31 ± 3.58	0.95

Data are presented as mean ± SD, median (range), or *n* (%). PACU, post-anesthesia care unit; p-RBC, packed red blood cell; RIPostC, remote ischemic postconditioning; SD, standard deviation.

Additional intraoperative and postoperative parameters, including PACU time, operative procedure type, resection indications, estimated blood loss, transfusion fluid volume, urine output, packed red blood cell (p-RBC) transfusion rate, intraoperative vasoactive drug use rate, and postoperative hospital stay, also showed no statistically significant differences between the groups (all *p* > 0.05, Table 2).

### Postoperative liver function assessment

**Figure 2:**
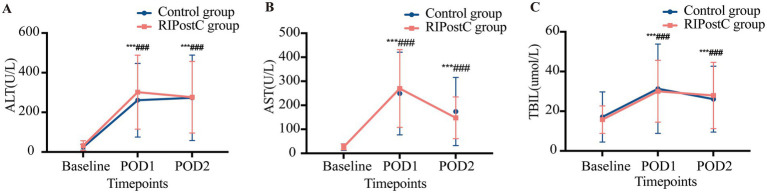
Liver function variables at baseline, POD1, and POD2. **(A)** ALT levels; **(B)** AST levels; **(C)** TBIL levels. The results are presented as means ± SD, and repeated measures analysis of variance was used to compare the results. There was no significant difference in ALT, AST, and TBIL levels between the two groups on POD1 and POD2 (ALT: (*p* = 0.73, *p* = 1.00); AST: (*p* = 0.77, *p* = 0.61; TBIL: (*p* = 0.99, *p* = 0.95). The levels of serum ALT, AST, and TBIL on POD1 and POD2 in both groups were significantly higher than those at the preoperative baseline, and the differences were statistically (*p* < 0.001). In the Control group vs. Baseline: ^*^p < 0.05, ^**^p < 0.01, ^***^p < 0.001; In the RIPostC group vs. Baseline: ^#^*p* < 0.05, ^##^*p* < 0.01, ^###^*p* < 0.001. ALT, alanine transaminase; AST, aspartate transaminase; TBIL, total bilirubin; RIPostC, remote ischemic postconditioning; POD, postoperative day; SD, standard deviation.

Serum ALT, AST, and TBIL levels increased significantly on postoperative days 1 and 2 compared to baseline levels in both groups (all *p* < 0.001, Figure 2).

However, statistical analysis using a two-way repeated-measures ANOVA indicated that there were no significant differences in ALT, AST, and TBIL levels between the two groups at either postoperative time point ([ALT:*p* = 0.73, *p* = 1.00]; [AST:*p* = 0.77, *p* = 0.61]; [TBIL: *p* = 0.99, *p* = 0.95]). These results indicate that the intervention did not significantly alter early postoperative liver function compared to the control group (Table 3; Figure 2).

**Table 3:** Biochemical markers of liver function at each time point.

Variables	Time points	Control group (*n* = 36)	RIPostC group (*n* = 36)	*p*-value
ALT (U/L)	Baseline	24.1 ± 15.1	32.0 ± 24.7	0.29
	POD1	261.1 ± 185.7	301.9 ± 186.4	0.73
	POD2	273.2 ± 215.5	276.4 ± 180.4	1.00
AST (U/L)	Baseline	26.0 ± 13.6	26.8 ± 12.2	1.00
	POD1	249.1 ± 172.1	269.8 ± 161.7	0.77
	POD2	174.0 ± 141.9	147.8 ± 86.9	0.61
TBIL (μmol/L)	Baseline	17.1 ± 12.6	15.8 ± 6.9	0.93
	POD1	31.3 ± 22.5	30.1 ± 15.6	0.99
	POD2	26.1 ± 16.6	28.0 ± 16.7	0.95

Data are presented as mean ± SD. ALT, alanine transaminase; AST, aspartate transaminase; TBIL, total bilirubin; RIPostC, remote ischemic postconditioning; POD, postoperative day.

## Discussion

This study is the first randomized controlled clinical trial to evaluate whether Remote ischemic postconditioning (RIPostC) exerts a protective effect on liver function in patients undergoing laparoscopic partial hepatectomy with hepatic portal occlusion. Our findings indicate that RIPostC did not significantly reduce the incidence of Hepatic ischemia–reperfusion injury (HIRI) or shorten postoperative hospital stay. These results are in contrast with preclinical studies suggesting that RIPostC enhances antioxidant responses and reduces hepatocyte apoptosis and liver injury (24). However, our findings are consistent with a meta-analysis of 459 hepatectomy patients from seven studies (25), which concluded that remote ischemic preconditioning (RIPC) failed to mitigate liver injury after hepatectomy. Both of which concluded that remote ischemic preconditioning (RIPC) failed to mitigate liver injury after hepatectomy. The inconsistency between experimental and clinical findings suggest that the protective effects of remote ischemic conditioning (RIC) may be context-dependent, highlighting the need for further multicenter, large-sample studies to clarify its clinical utility.

### Potential factors influencing the lack of RIPostC efficacy

Despite strong preclinical evidence, our study did not observe the anticipated benefits of RIPostC. Several factors may contribute to the absence of a clear protective effect of RIPostC in our human study.

Anesthetic interference: propofol and remifentanil anesthesia, both used in our study, have been shown to enhance hepatocyte tolerance to ischemia–reperfusion injury, potentially masking any additional protective effects of RIPostC (26, 27).

Prolonged hepatic portal Occlusion: Although a Pringle time of 30 min is considered safe (4, 5), the actual ischemic duration in both groups exceeded this threshold, possibly exacerbating irreversible hepatic damage and limiting RIPostC’s effectiveness.

Impact of cirrhosis: Some patients in our study had pre-existing cirrhosis, which may increase hepatocyte sensitivity to IRI (16). Prior studies have suggested that RIPC is less effective in cirrhotic patients (28), potentially due to hepatic sinusoidal endothelial cell dysfunction, reduced vascular endothelial growth factor (VEGF) and nitric oxide (NO) release, and impaired activation of RIPostC’s endogenous protective pathways (29).

Postoperative blood samples were collected only on days 1 and 2. As a result, potential delayed peaks in TBIL or cytokines may have been missed, which might underestimate the protective effects of remote ischemic postconditioning.

Perioperative hepatoprotective medications: Routine administration of hepatoprotective agents such as glycyrrhizic acid and glutathione may have stabilized hepatocyte membranes and reduced oxidative stress, thereby confounding the assessment of RIPostC’s efficacy (30, 31).

### Potential mechanisms and future directions

While animal studies suggest that RIPostC mitigates HIRI by modulating inflammatory and oxidative stress responses (32), its clinical effectiveness remains uncertain. RIPostC has been shown to downregulate inducible nitric oxide synthase (i-NOS), nuclear factor-κB (NF-κB), tumor necrosis factor-alpha (TNF-*α*), and interleukin-6 (IL-6), while enhancing antioxidant defenses via superoxide dismutase (SOD) and glutathione (GSH) (32). However, these molecular markers were not measured in our study, limiting our ability to explore potential protective mechanisms. Future studies should incorporate cytokine profiling and histopathological analyses to comprehensively evaluate RIPostC’s impact on liver function (33). At present, it has been determined that RIPostC can affect HIRI through the modulation of signaling pathways; for example, RIPostC can promote the phosphorylation of protein kinase B (AKT) and mitogen-activated protein kinase (MAPK). And the protective effect against liver I/R was abolished. RIPostC improves HIRI by activating the phosphatidylinositol 3-kinase (PI3K)/AKT signaling pathway and increasing Akt phosphorylation expression (24). In addition, some cell regulators that affect the development of IRI by RIPostC have been identified. These include heme oxygenase-1 (HO-1), hypoxia-inducible factor-1α (HIF-1α) and B-cell lymphoma-2 (Bcl-2), and these factors play a vital role in the protection of RIPostC organ cells from reperfusion injury (20, 24, 32). Additionally, the complex cellular interaction with the human liver further complicates the translation of findings from preclinical experiments. In this regard, recent single-cell transcriptome atlases of human liver have provided valuable insight into the remarkable heterogeneity of hepatic cell population (34–36). This cellular and molecular diversity underscores the complexity of human ischemia–reperfusion injury, and may partially explain why the robust benefits of RIPostC observed in animal models have not been consistently reproduced in clinical settings.

These potential mechanisms will provide valuable reference for clinical and scientific research, as an important research direction to further explore the mechanism of RIPostC in the prevention and treatment of HIRI, and better prevent or intervene in HIRI in the future. Moreover, the optimal RIPostC protocol remains undefined. Although our trial adopted a single-cycle regimen, which limits the generalizability of the findings to particular scheme. Chen et al. (19) demonstrated that prolonged RIPostC (5 cycles over 10–14 days) effectively reduced infarct size and improved neurological function in stroke patients. In contrast, a shorter protocol (4 cycles over 4 days) failed to show similar benefits (37). This heterogeneity in outcomes highlights that protocol design-including timing, duration, and frequency-is critical, and the lack of optimization at the design stage may have also contributed to the negative result in our study.

Although RIPostC was ineffective in our study, pharmacological interventions have been explored as alternative protective strategies for mitigating HIRI. For example, dexmedetomidine, an α_2_-adrenergic receptor agonist, has been shown to exert protective effects against HIRI by reducing inflammatory responses and oxidative stress. Liang Zhang et al. (38) found that intraoperative low-dose dexmedetomidine administration was associated with reduced HIRI in pediatric deceased liver transplantation. This suggests that anesthetic and sedative agents may have a more significant role in hepatic protection than ischemic conditioning techniques. The potential for combining RIPostC with pharmacological agents such as dexmedetomidine warrants further investigation. Future studies might therefore consider combining RIPostC with pharmacological agents such as dexmedetomidine to enhance potential benefits.

In addition, in our center the postoperative use of glycyrrhizic acid and glutathione was based on routine clinical practice rather than a strict protocol and was therefore not stratified in the present analysis. This could have introduced a confounding effect and potentially masked a small benefit of.

RIPostC, further underscoring the complexity of evaluating liver-protective strategies in clinical settings. Moreover, the challenge of finding effective therapeutic strategies for HIRI is evident in studies on Cyclosporine A, as demonstrated byJoshua Hefler et al. (39), who showed that Cyclosporine A did not mitigate liver ischemia/reperfusion injury in an ex vivo porcine model of donation after circulatory death. This highlights the broader issue that many pharmacological and conditioning interventions have failed to translate into significant clinical benefits. These findings underscore the necessity of identifying novel approaches that can provide consistent and reproducible liver protection during surgery.

### Study limitations

Our study has several limitations. First, we did not assess inflammatory markers such as TNF-*α*, IL-6, and IL-10, which may play a key role in the protective mechanisms of RIPostC (33). Second, liver function assessment was monitored only for 48 h, primarily through transaminase levels, more sensitive indicators such as total bilirubin (TBIL), which peaks around postoperative day 5, were not evaluated (4, 40). Given that our study only monitored liver function for 48 h postoperatively, we may have underestimated RIPostC’s long-term effects. Third, major postoperative complications subanalyses were not assessed due to limited sample size, which could have obscured potential benefits. Finally, as a single-center study with a relatively few participants, our findings are subject to potential false negatives. Future studies should optimize the RIPostC protocol (e.g., multi-cycle regimens), extend follow-up, and employ multi-center designs with larger cohorts to provide more definitive evidence.

## Conclusion

In summary, this randomized controlled trial demonstrated that a single-cycle regimen RIPostC did not significantly mitigate hepatic ischemia–reperfusion injury or improve postoperative liver function in patients undergoing laparoscopic hepatectomy. These results, together with prior mixed evidence, indicate that RIPostC cannot yet be considered definitively ineffective in liver resection, but its clinical benefits are likely protocol-dependent and potentially restricted to specific patient populations. While RIPostC remains a promising, non-invasive strategy, its clinical efficacy requires confirmation through multi-center trials with larger cohorts, longer follow-up, and optimized protocols, including multi-cycle regimens or adjunctive pharmacological approaches.

## Data Availability

The original contributions presented in the study are included in the article/supplementary material, further inquiries can be directed to the corresponding author.
